# Study of Construction and Performance on Photoelectric Devices of Cs–Pb–Br Perovskite Quantum Dot

**DOI:** 10.3390/ma14216716

**Published:** 2021-11-08

**Authors:** Shiyu Ma, Yan Lu, Bo Wang, Jinkai Li

**Affiliations:** 1 School of Materials Science and Engineering, University of Jinan, Jinan 250022, China; mashiyu1212@163.com (S.M.); mse_wangb@163.com (B.W.); 2 College of Mechanical and Electrical Engineering, Hainan Vocational University of Science and Technology, Haikou 571126, China; mly271515@163.com

**Keywords:** Cs–Pb–Br-based QDs, Gd_2_O_3_, Eu red phosphor, electric current stability, PLD technology

## Abstract

White LEDs were encapsulated using Cs_4_PbBr_6_ quantum dots and Gd_2_O_3_:Eu red phosphor as lamp powder. Under the excitation of a GaN chip, the color coordinates of the W-LED were (0.33, 0.34), and the color temperature was 5752 K, which is close to the color coordinate and color temperature range of standard sunlight. The electric current stability was excellent with an increase in the electric current, voltage, and luminescence intensity of the quantum dots and phosphors by more than 10 times. However, the stability of the quantum dots was slightly insufficient over long working periods. The photocatalytic devices were constructed using TiO_2_, CsPbBr_3_, and NiO as an electron transport layer, light absorption layer, and catalyst, respectively. The Cs–Pb–Br-based perovskite quantum dot photocatalytic devices were constructed using a two-step spin coating method, one-step spin coating method, and full PLD technology. In order to improve the water stability of the device, a hydrophobic carbon paste and carbon film were selected as the hole transport layer. The TiO_2_ layer and perovskite layer with different thicknesses and film forming qualities were obtained by changing the spin coating speed. The influence of the spin coating speed on the device’s performance was explored through SEM and a J–V curve to find the best spin coating process. The device constructed by the two-step spin coating method had a higher current density but no obvious increase in the current density under light, while the other two methods could obtain a more obvious light response, but the current density was very low.

## 1. Introduction

In the field of lighting display, LEDs, LCDs, and OLEDs are the main devices used presently. As a basic light-emitting device, LEDs have a wide range of application in household lighting [1,2,3,4], traffic signals, landscape decoration, and other fields. White LED devices usually adopt three packaging methods: a red, green, and blue chip combination; red, green, and blue chip activated phosphor; blue chip activated red, green, and blue phosphor. Compared with the other two packaging methods, the Blu-ray chip-excited red–green phosphor packaging method has the advantages of having a simple process, good stability, and low cost. However, as a typical green light type of quantum dots, the research on lead bromide-based perovskite quantum dots is less than that in the field of LED packaging.

The perovskite quantum dots with a Cs–Pb–Br structure have the advantages of small size, high quantum efficiency, wide color gamut, narrow half-peak width, and high carrier mobility. Compared with traditional phosphors, perovskite quantum dots are more energy saving and environmentally friendly and more suitable for the production of small-size or mini-LEDs. The stability of Cs_4_PbBr_6_ perovskite quantum dots is better than that of CsPbBr_3_ [5,6,7,8,9,10]. Therefore, Cs_4_PbBr_6_ perovskite quantum dots have high application value in encapsulated white LED devices.

In this paper, Cs_4_PbBr_6_ perovskite quantum dots, red phosphors, and LED chips were used to encapsulate white LED, and the performance of the devices was studied by EL spectrum and color coordinates. TiO_2_ as the electron transport layer, CsPbBr_3_ perovskite for the light absorption layer, carbon paste and carbon film for the holes transport layer, and an NiO catalyst layer were used. A photocatalytic device based on a multilayer thin-film solar cell structure was constructed using the solution spin coating method and pulsed laser deposition technology [11,12,13,14,15,16,17]. The effects of the spin coating rate on the TiO_2_ layer and perovskite layer are discussed, while the thickness and film quality of the TiO_2_ layer and perovskite layer on the performance of the device were analyzed.

## 2. Experimental Section

### 2.1. Materials and Chemicals

Cesium bromide (CsBr, Macklin, 99.999%), lead bromide (PbBr_2_, Macklin, 99.99%), oleylamine (OAm) (C_18_H_37_N, Macklin, 80–90%), oleic acid (OA, Macklin, AR), N-dimethylformamide (DMF, Macklin, ≥99.5%), methylbenzene (C_6_H_5_CH_3_, Yantai Yuandong Fine Chemical Co., Ltd. (Yantai, China), ≥99.5%), gadolinium nitrate (Gd(NO_3_)_3_, Macklin, 99.5%), titanium diisopropoxide bis(acetylacetonate) (Aladdin, 75%).

### 2.2. Characterization

The morphology of the precursor and resultant products were collected via field-emission scanning electron microscope (FE-SEM, QUANTA FEG 250, FEI Co., Waltham, MA, USA) with an acceleration voltage of 10 kV. The electrochemical performance of the samples was tested on an electrochemical workstation (Zahner/Zennium, Bavaria, Germany). The PLE and PL spectra were obtained using a Fluorescence Spectrophotometer (FP-6500, JASCO Co., Tokyo, Japan) at room temperature equipped with a Φ60 mm integrating sphere (ISF-513, JASCO, Tokyo, Japan) and a 150 W Xe-lamp as the excitation source. The optical performances for all samples were conducted under identical conditions with a slit breadth of 5 nm. The specimen was excited with a selected wavelength, and the intensity of the intended emission was recorded as a function of elapsed time after the excitation light was automatically cut-off using a shutter.

### 2.3. Synthesis of Cs_4_PbBr_6_

Both 0.0213 g (0.1 mmol) CsBr and 0.0368 g (0.1 mmol) PbBr_2_ were dissolved in 5 mL DMF solution, which contained 30 μL OAM and 0.5 mL OA. After ultrasonic dispersion and stirring for 15 min, the quantum dot precursor solution was obtained. The precursor solution 0.4 mL was injected into 5 mL of toluene solution, which was violently stirred continuously. A reaction occurred after 5 s, and the yellow–green colloid solution was formed and Cs_4_PbBr_6_ quantum dots were obtained. The colloid solution was placed in a centrifuge tube and centrifuged at 12,000 RPM for 5 min. After being washed twice with toluene, the precipitation was dried in a vacuum oven at 60 °C for 24 h.

### 2.4. Synthesis of Gd_2_O_3_:Eu Red Phosphor

Accurate weights of Gd_2_O_3_ and Eu^3+^ phosphors according to the stoichiometric ratio of 1:0.05. Then, 2.85 mL Gd(NO_3_)_3_ solution and 0.75 mL Eu (NO_3_)_3_ solution were added to a 250 mL beaker, stirred for 10 min after adding a suitable amount of deionized water, adding suitable amount of urea and stirring for 10 min to ensure it fully dissolved. The mixed solution was heated up to 90 °C quickly and maintained for 2 h at 90 °C until the reaction was complete.

The precipitate was obtained by centrifugation when the reaction solution was cooled to room temperature. The precipitate was cleaned twice with distilled water and anhydrous ethanol and dried at 80 °C. The precursor powder was calcined at 800 °C with a heating rate of 5 °C/min and a holding time of 4 h. The atmosphere of the calcination was air. After calcination, Gd_2_O_3_: Eu^3+^ phosphors were obtained.

### 2.5. Encapsulation of White LED

Bothe 0.005 g Cs_4_PbBr_6_ powder and 0.035 g Gd_2_O_3_:Eu red phosphor were placed in an agate mortar. They were fully ground and mixed. Then, 0.4 g of silicone resin A glue was added to the stir and mixed, after which 0.05 g of silicone resin B glue was added to lay the mixed phosphor on the GaN chip. After the removal of bubbles, the solidification was accelerated by heating. Next, 0.005 g Cs_4_PbBr_6_ powder and 0.035 g Gd_2_O_3_:Eu red phosphor were fully ground and mixed in an agate mortar, and 0.4 g of silicone resin A glue was added. After stirring and mixing, 0.05 g of silicone resin B glue was added. The mixed phosphor was tiled on the GaN chip and heated to accelerate its solidification after removing the bubbles.

### 2.6. Photoelectric Catalysis Devices Based on CsPbBr_3_ by Two Step Spin Coating

Photocatalytic devices based on CsPbBr_3_ are similar to multilayer solar cells or Q-LED devices. The glass substrate containing fluorine-doped tin oxide (FTO) was surface etched on and was cut into a 3 cm × 3 cm^2^, and then ultrasonic washed with acetone, methanol, and deionized water for 15 min. It was dried in N_2_ atmosphere and then exposed to UV–ozone for 15 min [18,19].

Then, 0.5 mL titanate was dissolved in 5 mL ethanol and spin-coated for 50 s at 2000–4000 RPM twice, followed by annealing at 500 °C for 60 min to form the TiO_2_ electron transport layer. Lead bromide was dissolved in anhydrous DMF (1 mmol/mL) and cesium bromide was dissolved in methanol (0.1 mmol/mL), which was spin-coated for 30 s at 2000–4000 RPM successively and then vacuum annealed at 300 °C for 30 min to form the perovskite layer. The carbon paste was scraped onto the perovskite layer by hand, and the graphite sheet was cut into 3 cm × 2.2 cm and pasted on the carbon paste. The carbon paste was annealed by vacuum at 120 °C for 60 min in order to solidify the film. The catalyst layer was deposited using the pulsed laser deposition (PLD) technique and annealed in vacuum at 200 °C for 120 min. Finally, the device was encapsulated with UV drops.

### 2.7. Photoelectric Catalysis Devices Based on CsPbBr_3_ by One Step Spin Coating

Both 0.0213 g (0.1 mmol) CsBr and 0.0440 g (0.12 mmol) PbBr_2_ were dissolved in 5 mL DMF that contained 30 μL OAM and 0.5 mL OA. The solution was stirred to dissolve all the drugs and the precursor solution was obtained after 15 min. The precursor solution (0.4 mL) was injected into a vigorously agitated toluene solution (5 mL). After reaction for 5 s, the colloidal solution was immediately centrifuged at 12,000 RPM for 5 min. The supernatant was discarded, the precipitation was dispersed in toluene for centrifugation, and CsPbBr_3_ quantum dots were obtained.

After the FTO glass was treated, the titanate ethanol solution was spin-coated 50 s at 3000 RPM and repeated twice, and the TiO_2_ electron transport layer was formed after annealing at 500 °C for 60 min. The toluene solution of CsPbBr_3_ quantum dots was spin-coated for 60 s at 3000 RPM and repeated twice. The solution was annealed in a vacuum at 100 °C for 60 min. The remaining steps were consistent with Section 2.4.

### 2.8. Photoelectric Catalysis Devices Prepared by PLD Technique

Low-pressure pulsed laser deposition (PLD) was used for the TIO_2_ layer deposition. The TiO_2_ layer was formed after annealing at 500 °C for 1 h with a pulse frequency of 30,000 at 5 Hz and 100 mJ.

The lead bromide target and cesium bromide target were also deposited at the frequency of 5 Hz, 100 mJ and energy of 20,000 pulses, and the perovskite layer was formed by vacuum annealing at 300 °C for 30 min. The NiO layer was grown on the nickel oxide target with 150 mJ energy and a 5 Hz pulse frequency of 10,000, and annealed in a vacuum at 200 °C for 120 min.

## 3. Results and Discussion

In order to study the application value of Cs_4_PbBr_6_ quantum dots in lighting technology, we mixed Cs_4_PbBr_6_ quantum dots and red Gd_2_O_3_:Eu phosphor as lamp powder, encapsulated them on GaN chip at 380 nm using silicone resin, and assembled simple white light-emitting diodes (W-LEDs).

**Figure 1:**
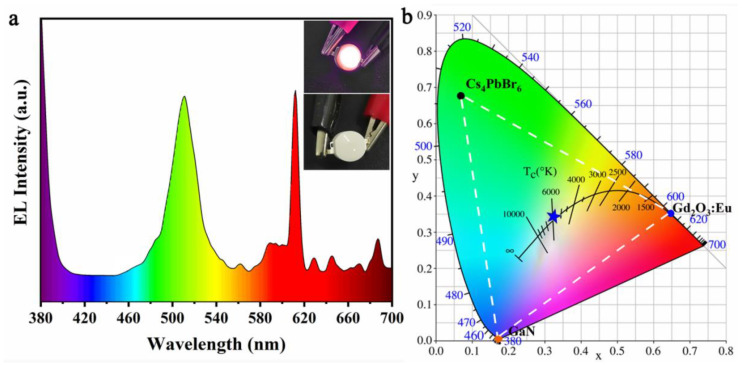
(**a**) EL spectrum of W-LED illustrated with photos after and without power on; (**b**) color coordinate diagram of W-LED.

Figure 1a shows the EL spectrum of a W-LED. Three emission peaks are intuitively displayed in the spectrum. The emission peak at 380 nm corresponded to the emission peak of the chip, the emission peak at 511 nm was the emission peak of Cs_4_PbBr_6_ quantum dots excited by the chip. The satellite peak at 612 nm was the emission peak of Gd_2_O_3_:Eu. The illustration in Figure 1a shows the emission photos of the W-LED when it was energized and non-energized. It can be seen that under the excitation of the blue chip in the energized state, the mixed light of green quantum dots and red fluorescent powder presents white light emission.

According to the emission of W-LEDs, their color coordinates are depicted, respectively, in CIE chromaticity diagram. As shown in Figure 1b, the color coordinates of Cs_4_PbBr_6_ quantum dots and Gd_2_O_3_:Eu red phosphor were (0.07, 0.68) and (0.65, 0.35), respectively. The color coordinates of the encapsulated W-LED were (0.33, 0.34), and its color temperature was calculated to be 5752 K, which is close to the color temperature range of standard sunlight (5200~5500 K).

**Figure 2:**
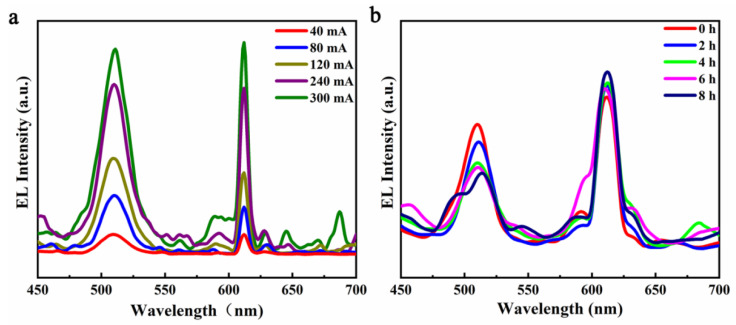
(**a**) EL curves of the W-LED under different current currents; (**b**) the EL curve of W-LED varied with time.

In the practical application of the W-LED, its current fluctuated generally. For this reason, the EL curves of the lamp beads at 40 mA to 300 mA were tested, as shown in Figure 2a. As the current increases from 40 mA to 300 mA, the voltage at both ends of the lamp bead’s increases from 3.06 to 3.31 V, and the luminescence intensity of both quantum dots and phosphors increased more than 10 times. This indicates that the W-LED based on Cs_4_PbBr_6_ had good current and voltage stability. In addition, under the current of 3.1 V and 40 mA, the changes in the emission intensity of the W-LED under the energized state for 8 h were tested as shown in Figure 2b. It can be seen that the luminescence intensity of Cs_4_PbBr_6_ quantum dots gradually decreased with the extension of time. After 8 h of aging, the luminescence intensity of the Cs_4_PbBr_6_ quantum dots only retained 65.43% of their initial value, which indicates that the stability of the quantum dots was slightly insufficient. This may have been caused by the lack quantum dots’ lack of optical stability, which needs to be further optimized.

The long-term stability of the quantum dot-based LED was poorer than the Gd_2_O_3_:Eu red phosphor LED, which was determined by the characteristics of the quantum dots themselves. Perovskite materials will degrade under the influence of air, water, thermal radiation, and light radiation and will then lose their original photoelectric properties. Roda et al. found that O_2_ could combine with photo-generated electrons to cause PL quenching without defect guidance [20]. The influence of light on perovskite materials was mainly reflected in two aspects: one is that photogenerated electrons will promote the destruction of oxygen on perovskite, the other is the photodegradation under the joint action of surface defects. When perovskite absorbs photons, it produces charge carriers, which may spread to the crystal surface. The defect acts as an electron trap on the crystal surface and the trap’s photocarriers, forming a local trap state. With the extension of illumination time, the density of the trap gradually increases, which further disrupts the crystal structure and generates an electric field, further intensifying the coupling between carriers and lattice, ultimately leading to lattice distortion and structural degradation of perovskite [21]. Gd_2_O_3_:Eu red phosphor is a kind of phosphor in essence, which is superior to the perovskite quantum dot in stability.

**Figure 3:**
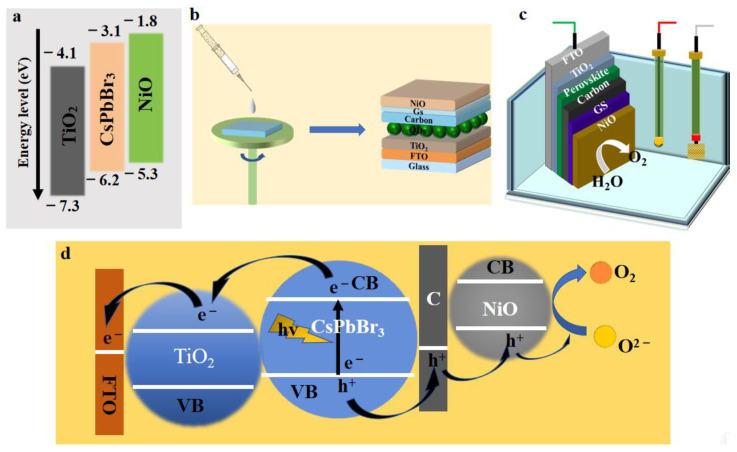
(**a**) Energy level structure diagram of TiO_2_, CsPbBr_3_, and NiO; (**b**) schematic diagram of photocatalytic device constructed by solution method; (**c**) schematic diagram of a photochemical test of a perovskite-type optoelectronic device using a standard three-electrode system with the photocatalytic device backlit from the FTO side; (**d**) diagram of photocatalytic activity mechanism of CsPbBr_3_ QDs photoelectric devices.

In order to explore the application potential of CsPbBr_3_ perovskite materials in photoelectric decomposition water, TiO_2_ and NiO were selected as the electron transport layer (ETM) and catalytic materials, respectively. According to the principle of energy level matching, based on the determination of CsPbBr_3_ energy levels [22,23,24,25], the schematic diagram of their energy level structure was drawn as shown in Figure 3a.

Perovskite material degrades due to the hydrolytic reaction in contact with water, which will lead to device failure [26]. Hydrolysis reactions must be carried out in solution. Therefore, in order to improve the water stability of the device, a combination of carbon paste and carbon film was used as the hole transport layer (HTM). As a good conductor, carbon has a very strong ability for hole transport, and the dense carbon film was a hydrophobic material. Such a hole transport layer provided both waterproof protection and hole transmission.

The design and manufacture of a photocatalytic device with a standard multilayer thin-film solar cell structure is shown in Figure 3b. Using titanate as precursor to spin coating on the FTO glass substrate as electron transport layer, the antisolvent spin coating method was used to deposit perovskite on the top of TiO_2_ layer. Carbon paste was used to cover the surface of CsPbBr_3_ as the hole transport material layer, and a graphite conductive sheet was used as the encapsulation and conducting material. Ultrathin nickel oxide layer was deposited by PLD as the outermost layer of the catalytic device. Finally, the device was encapsulated with UV curable adhesive. Each layer should be as flat as possible with minimal porosity.

As shown in Figure 3c, the photocatalytic device was completely immersed in the electrolyte to construct a photochemical cell with reference electrode and counter-electrode. As shown in Figure 3d, under sunlight (from backlight), photogenerated electrons in CsPbBr_3_ were transferred by the TiO_2_ layer to the FTO for collection, and then flowed through the external circuit to the opposite electrode, where the water-receiving electrons on the surface of the opposite electrode are reduced to produce hydrogen. The holes migrated through the carbon layer to the NiO layer to produce oxygen by oxidizing the sacrifice electrolyte (KOH solution) in the solution. Importantly, the NiO layer at the top not only acted as a physical barrier for electrolyte penetration into the active perovskite layer but also acted as an effective oxidation catalyst with generated cavities [27].

**Figure 4:**
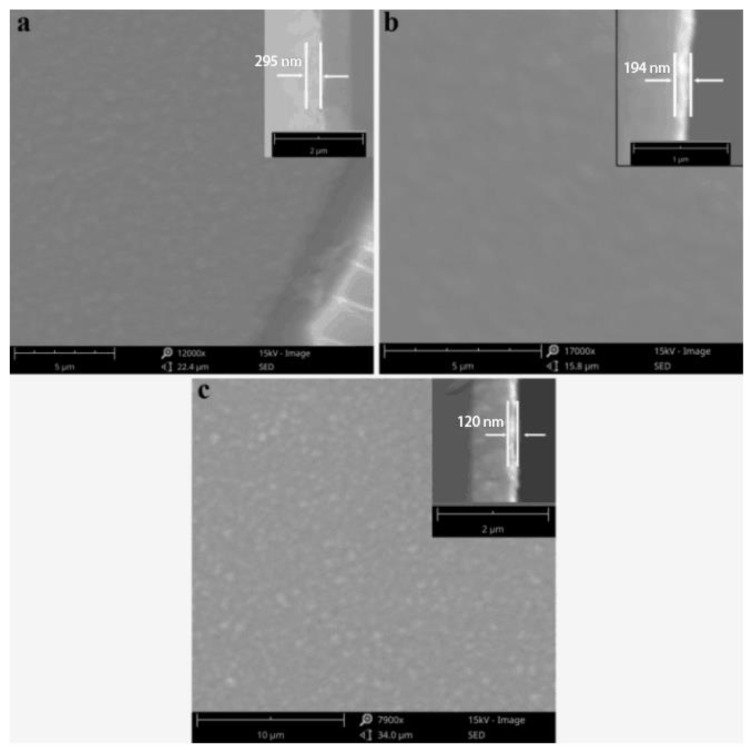
SEM images of front and cross-section of the TiO_2_ layer at different spin-coating speeds: (**a**) 2000 RPM, (**b**) 3000 RPM, (**c**) 4000 RPM; The inset shows the section scan at the corresponding spin-coating speed.

We explored the spin coating process of the TiO_2_ layer at different spin coating speeds as shown in Figure 4. After annealing at 500 °C, dense TiO_2_ was formed by spin-coating the titanate/ethanol solution at 2000, 3000, and 4000 RPM, respectively. As can be seen from the SEM image of cross-section in Figure 4, with the increase in the spin-coating rate, the thickness of the TiO_2_ film decreased from 295 to 120 nm. At the same time, it can be seen from SEM surface scan that dense TiO_2_ films were formed at the three rotational speeds, but the films formed at 2000 RPM and 4000 RPM were slightly rough. Due to the low rotational speed at 2000 RPM, agglomerated small particles existing in titanate/ethanol solution will still remain on the film after spinning. TiO_2_ film remained rough after annealing. Because the surface of the FTO glass was rough, and the TiO_2_ film formed at 4000 RPM had a small thickness that was not enough to make up for the uneven surface of the FTO; thus, a rough TiO_2_ film formed. However, the film formed at 3000 RPM was relatively thick, and the high rotating speed can ensure that large particles will not remain, so a smoother, compact and flat TiO_2_ film is formed.

**Figure 5:**
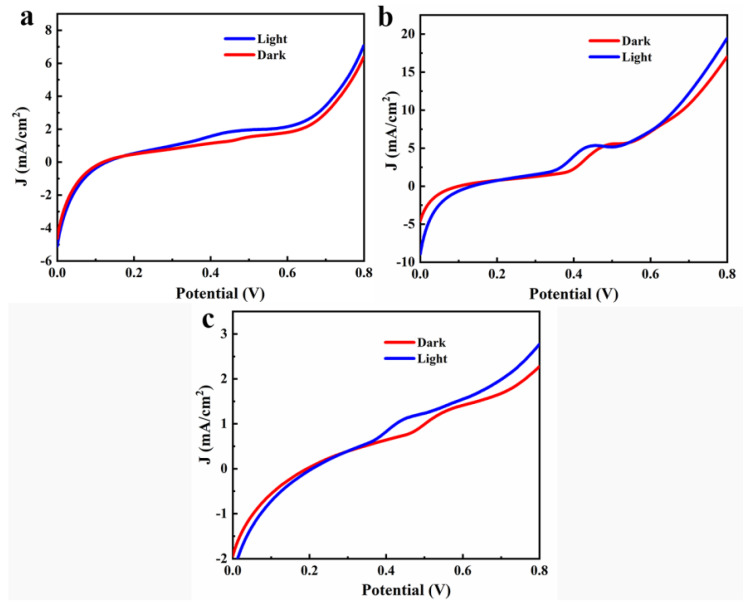
J–V curves of the TiO_2_ layer at different spin-coating speeds: (**a**) 2000; (**b**) 3000; (**c**) 4000 RPM.

Its J–V curve was obtained as shown in Figure 5. The current density of the devices with three spin coating speeds increased to a certain extent after illumination (10.99%, 14.30%, 21.80%, respectively with the increase in rotation speed. This was due to the dense and flat TiO_2_ film, which obtained the highest current density and higher photocurrent enhancement at 3000 RPM. In addition, we found that there was a certain current density when the voltage was 0 V, which may be attributed to the transfer of electrons and holes in the perovskite layer under the irradiation of ambient light [18]. The current density variation between 0.35 and 0.55 V was attributed to the REDOX potential of the NiO hydrate NiOOH.

**Figure 6:**
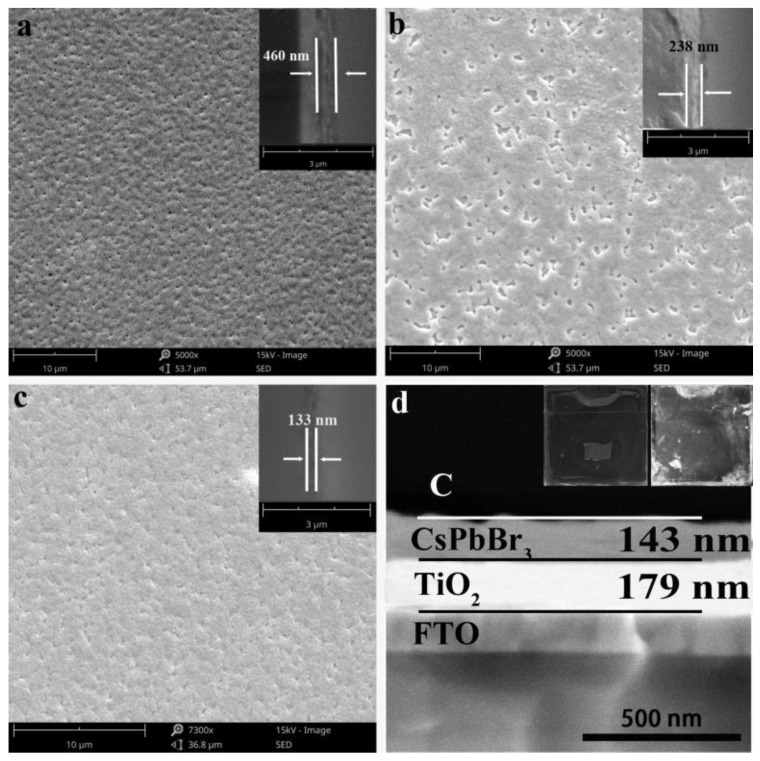
SEM images of front and cross-section of CsPbBr_3_ layer at different spin-coating speeds: (**a**) 2000; (**b**) 3000; (**c**) 4000 RPM. The inset shows the section scan at the corresponding spin-coating speed. (**d**) A cross-sectional scan of the photocatalytic device illustrated with digital photos of the front and back of the packaged device.

The spinning coating technology for the CsPbBr_3_ layer is further discussed below. The CsPbBr_3_ layer was prepared by a two-step spin coating process. The first step was spin-coating the PbBr_2_ DMF solution, and the second step was spin-coating the CsBr methanol solution. The CsPbBr_3_ layer was formed after annealing. The spin-coating speed of PbBr_2_ and CsBr was adjusted at the same time to obtain CsPbBr_3_ layers with different characteristics. Its performance characteristics were characterized by SEM images. As shown in Figure 6, compared with TiO_2_ layer, the film forming quality of CsPbBr_3_ layer was relatively poor, and the porosity was higher. At 2000 RPM, the thickness reached 460 nm. The higher layer thickness made the surface of the CsPbBr_3_ layer uneven and full of small pores. The thickness of the perovskite layer at 3000 RPM was approximately 238 nm, but the pore size of the perovskite layer is increased, although the number of pores is reduced compared with that at 2000 RPM. Only when the rotating speed was increased to 4000 RPM, a relatively smooth, dense, and low-porosity CsPbBr_3_ layer could be obtained, and the thickness of the layer was 133 nm. In combination with the discussion on the TiO_2_ layer technology, we used a spinning speed of 3000 RPM to obtain the TiO_2_ layer, and a spinning speed of 4000 RPM was used to obtain the CsPbBr_3_ layer. After scraping and coating the carbon paste and sticking the graphite sheet, the obtained device was scanned again for the cross-section as shown in Figure 6d. The thickness of TiO_2_ layer and CsPbBr_3_ layer obtained in this study were 179 and 143 nm, respectively. A digital photo of the encapsulated device is shown in Figure 6d, where the rectangular window with no glue in the middle is the test surface, and the upper window is the electrode clamp.

**Figure 7:**
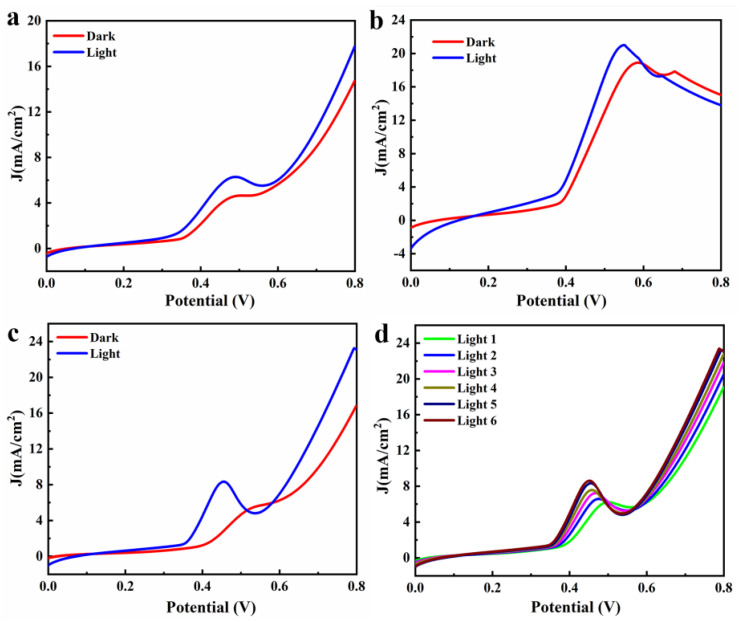
J–V curves of the CsPbBr_3_ layers at different spin-coating speeds: (**a**) 2000; (**b**) 3000; (**c**) 4000; (**d**) 4000 RPM. (**d**) J–V curves of the photocatalytic device obtained by spin coating at 4000 RPM under illumination.

The TiO_2_ layer was obtained by spin coating at 3000 RPM, and the perovskite layer was obtained at different spinning coating speeds. Using a 300 W xenon lamp as light source for continuous illumination. Under continuous lighting conditions, the electrochemical workstation automatically measured. The frequency was measured six times every 4 min. The J–V curves of the device are shown in Figure 7. The devices obtained at the three spinning speeds all had higher current densities. Under light condition, the current density of the devices obtained at 2000, 3000, and 4000 RPM increased by 24.49%, 11.18%, and 38.27%, respectively. Because the layer of CsPbBr_3_ was relatively flat, the current density and the increase in the current density at 4000 RPM were the highest. Due to the large pore area of the perovskite layer obtained at 3000 RPM, the current density of the J–V curve decreased after 0.55 V, which led to the leakage phenomenon. As shown in Figure 7d, the J–V curve of the photocatalytic device obtained by spin-coating at 4000 RPM was tested many times under illumination. The current density increased first and then stabilized. This indicates that the activation of the CsPbBr_3_ layer took a certain time, and the best performance of the device could only be achieved after a period of illumination.

This was similar to the device obtained by the one-step method. PLD devices also had an obvious optical response. When the light source was turned on, the current density increased to 118.58%, but its current density was only 0.036 mA/cm^2^. The higher current density may be related to the dense perovskite deposits. The use of PLD laser deposition technology will form a very dense film but will also form a layer of plasma glow between the layers, blocking the migration of electrons between layers, resulting in a significant increase in electrical resistance.

**Figure 8:**
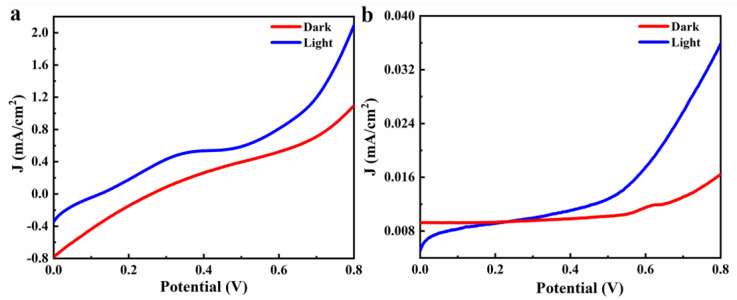
(**a**) J–V curves of the photocatalytic devices prepared by PLD technology; (**b**) J–V curves of the device obtained by replacing the perovskite layer with CsPbBr3 quantum electricity.

In addition, a one-step method (spin-coated pre-prepared CsPbBr_3_ quantum dot toluene solution) was also attempted to replace the two-step method to prepare perovskite quantum dot layers. The J–V curve of the device obtained is shown in Figure 8a. Compared with the two-step preparation process, the optical response of the device obtained by the one-step method was more obvious, and the current density under illumination increased by 90.80%, which was much higher than the 38.27% of the two-step method. However, the maximum current density was only 2.09 mA/cm^2^, which was much lower than 23.25 mA/cm^2^. This was because the spin-coating solution of the one-step coating method was the solution of the CsPbBr_3_ quantum dots. Perovskite quantum dots been formed, so the spin-coating quantum dots had a high content and dense film formation and, thus, had a high light response current density. In the two-step coating method, the reagents that can generate perovskite were rotated on glass substrate (FTO) two times. The formation of perovskite quantum dots requires imaging in the process of rotating coating. Due to the limited contact surface and uneven mixing of the two-step coating reagents, the quantum dots content of the perovskite layer that formed was low, and the film quality was inferior to that of the one-step coating method.

It may be that the interlayer resistance was high because the spin-coated QDs were not annealed at high temperature. We also tried to use PLD deposition to form TiO_2_ and CsPbBr_3_ layers, and the J–V curve obtained is shown in Figure 8b. This was similar to the devices obtained by the one-step method, the PLD devices also had obvious optical response. The current density increased to 118.58%, but the current density was only 0.036 mA/cm^2^. The higher rate of current density increase may be related to the dense perovskite deposits. Very dense thin films can be formed by laser deposition. At the same time, PLD deposition technology will form a layer of plasma glow between layers, which hinders the migration of electrons between layers also leads to a significant increase in electrical resistance.

## 4. Conclusions

The white LED was encapsulated by Cs_4_PbBr_6_ quantum dots and Gd_2_O_3:_Eu red phosphor as the light powder. Under the excitation of a GaN chip, the color coordinates of the W-LED was (0.33, 0.34), and the color temperature was 5752 K, which was close to the color coordinate and color temperature range of standard daylight. W-LED devices show excellent current stability. With the increase in current and voltage, the luminescence intensity of quantum dots and phosphors increased by more than 10 times. However, the stability of the quantum dots was slightly less than that of phosphors when the current was on for a long time.

At the rotating speeds of 2000, 3000, and 4000 RPM, the thickness of the spin-coated TiO_2_ layer decreased gradually. The film forming quality was the best at 3000 RPM, and the current density of the device was the highest at 3000 RPM. However, the increase in the current density increased with the increase in rotational speed. The thickness of CsPbBr_3_ layer decreased, and the film quality increased with the increase in rotating speed. The best film quality, the highest current density, and the current increase range were obtained at 4000 RPM. The best device performance can be obtained by preparing a TiO_2_ layer at 3000 RPM and a sCsPbBr_3_ layer at 4000 RPM.

The photocatalytic device was constructed by means of toluene solution of spin coated quantum dots and PLD technology. The current density of the device was greatly improved when the light source was turned on. The device constructed by PLD technology improved by 118.58%, but its current density was much lower than that of the device constructed by solution spin coating method. This indicates that the perovskite layer prepared by PLD technology was denser and the interlayer resistance was larger.

## Data Availability

Not applicable.
